# Selective DNAM-1 expression on small peritoneal macrophages contributes to CD4^+^ T cell costimulation

**DOI:** 10.1038/s41598-018-33437-4

**Published:** 2018-10-12

**Authors:** Eri Takenaka, Anh Van Vo, Yumi Yamashita-Kanemaru, Akira Shibuya, Kazuko Shibuya

**Affiliations:** 1 Department of Immunology, Faculty of Medicine, Tsukuba Advanced Research Alliance (TARA), University of Tsukuba, Tsukuba, Japan; 2 PhD Program in Human Biology, School of Integrative and Global Majors, Tsukuba Advanced Research Alliance (TARA), University of Tsukuba, Tsukuba, Japan; 3 Life Science Center for Survival Dynamics, Tsukuba Advanced Research Alliance (TARA), University of Tsukuba, Tsukuba, Japan

## Abstract

Mouse peritoneal macrophages consist of two subsets: large peritoneal macrophages (LPMs) and small peritoneal macrophages (SPMs), defined as CD11b^hi^F4/80^hi^ and CD11b^+^F4/80^lo^ cells, respectively. We reveal that SPMs, but not LPMs, have the ability to present antigens to naïve CD4^+^ T cells. Coculture of SPMs with naïve ovalbumin (OVA) specific CD4^+^ T cells (OT-II) in the presence of OVA peptide effectively induced CD4^+^ T cells priming. SPMs, but not LPMs, strongly express DNAM-1, an activating immunoreceptor. Although antigen uptake and processing were comparable between WT and DNAM-1-deficient SPMs, deficiency of DNAM-1 on SPMs or blockade of DNAM-1 and its ligand interaction impaired CD4^+^ T cells priming by SPMs. Furthermore, T and B cell responses in mediastinal lymph nodes of mice intraperitoneally immunized with trinitrophenyl (TNP)–OVA protein in Alum adjuvant were enhanced by intraperitoneally transferred wild-type, but not DNAM-1-deficient, SPMs. We propose that SPMs are functionally distinct from LPMs, and DNAM-1 plays a costimulatory role in antigen presentation by SPMs.

## Introduction

In the past decade, the importance of distinguishing heterogeneous populations of tissue macrophages on the basis of surface molecules and functions has been recognized^1,2^. In the mouse peritoneal cavity, macrophages are divided into two distinct subsets named large peritoneal macrophages (LPMs) and small peritoneal macrophages (SPMs)^3^. LPMs, which are defined as CD11b^hi^F4/80^hi^ and express low levels of MHC class II (MHCII), are an abundant macrophage subset and likely correspond to the typically characterized peritoneal macrophages, which express CD11b and F4/80. In contrast, SPMs, which are defined as CD11b^+^F4/80^lo^ and express high levels of MHCII, are a newly determined minor subset. LPMs and SPMs respectively account for >90% and approximately 3.5%–10% of peritoneal macrophages in a variety of mouse strains, including BALB/c and C57BL/6^3^. LPMs are suggested to be derived from yolk sac independently of hematopoiesis and to be maintained by self-renewal^4–6^; in addition, some reports have described the coexistence of a demand-driven replenishment pathway for LPMs from hematopoietic progenitors^6,7^. SPMs appear to be sustained through hematopoiesis, and are likely derived from inflammatory monocytes^7,8^. After inflammatory stimulation, inflammatory monocytes infiltrate into the peritoneal cavity and may differentiate into SPMs^3^.

Both SPMs and LPMs show phagocytic ability; however, phagocytic activity against bacteria^3^ and zymosan^9^ is higher in SPMs than LPMs, whereas phagocytic activity against apoptotic cells is lower in SPMs than LPMs^7^. LPMs produce abundant G-CSF, GM-CSF, and CXCL1 after *in vitro* LPS stimulation^7^, and they produce nitric oxide (NO) in response to LPS both *in vitro* and *in vivo* (1). A recent report showed that LPMs induce IgA production by B-1 cells through GATA6-dependent TGF-β production^8^. On the other hand, SPMs produce greater amounts of MIP-1α (CCL3) and TNFα than do LPMs in response to *in vitro* LPS stimulation^7^. SPMs produce NO after *in viv*o LPS stimulation, but not after *in vitro* LPS stimulation^3^. Data from the work of Cassado *et al*. suggest that SPMs also produce NO and IL-12 after infectious stimulation with *Trypanosoma cruz*i^9^. However, little else is known about the physiological functions of SPMs and the functional molecules in SPMs remain unclear.

The DNAX accessory molecule-1 (DNAM-1, also known as CD226) is a member of the immunoglobulin superfamily and is constitutively expressed on the majority of NK cells, CD8^+^ T cells, CD4^+^ T cells, monocytes, and platelets in both human and mouse^10,11^. CD155 (also known as poliovirus receptor [PVR], Necl-5, Tage4) and CD112 (also known as PRR-2 or nectin-2) are ligands for human and mouse DNAM-1^11–13^. CD155 and CD112 are broadly expressed on hematopoietic, epithelial, and endothelial cells in many tissues in human and mouse^13–15^. Interactions between DNAM-1 on NK cells or CD8^+^ T cells and CD155 or CD112 on target cells enhances cell-mediated cytotoxicity against target cells and cytokine production^12,13,16–19^; however, the expression profile and function of DNAM-1 on macrophages remain unclear.

Here, we investigated the functional difference between SPMs and LPMs and revealed that SPMs, but not LPMs, have the ability to present antigens to naïve CD4^+^ T cells. We further explored that DNAM-1 is highly expressed on SPMs, but not LPMs and play a costimulatory role in antigen presentation to CD4^+^ T cells by SPMs.

## Results

### SPMs have the ability to prime antigen specific CD4^+^ T cells

**Figure 1:**
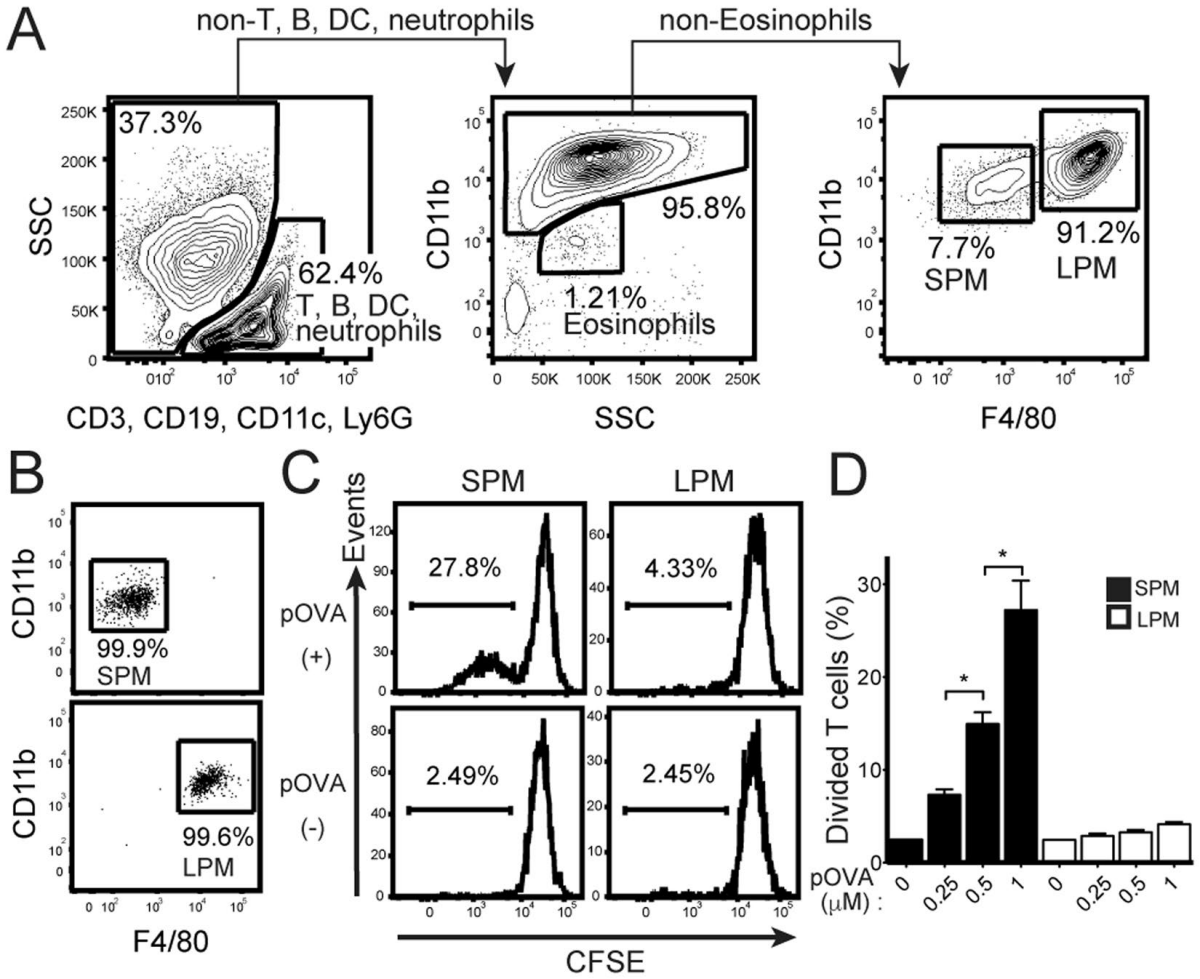
SPMs have the ability to present antigen to naïve CD4^+^ T cells. (**A**) Gating strategy of SPMs (CD11b^+^F4/80^−^) and LPMs (CD11b^hi^F4/80^hi^) in peritoneal cavity cells from naïve C57BL/6 mice. CD3^+^ T cells, CD19^+^ B cells, CD11c^+^ dendritic cells (DCs), Ly6G^+^ neutrophils, and CD11b^lo^SSC^hi^ eosinophils were excluded, and SPMs and LPMs were distinguished. Numbers indicate percentage of the total population in each region. (**B**) Sorting purity of SPMs (upper panel) and LPMs (lower panel). (**C**, **D**) CD62L^hi^CD44^dull^ CD4^+^ naïve T cells isolated from OT-II Tg mice were labeled with carboxyfluorescein succinimidyl ester (CFSE); and 2 × 10^4^ cells were cocultured with same number of SPMs or LPMs isolated from peritoneal cells of naïve mice, in the presence of pOVA at indicated doses. On day 5, the proliferation of CD11b negative-gated T cells was assessed by flow cytometry analysis of CFSE dilution. Histograms show representative data after culture with or without 1 µM pOVA (**C**). Bar graph shows the average of three wells for each of the indicated pOVA concentrations (**D**); error bars indicate SEM. *P < 0.05. Data is representative of two independent experiments.

To investigate the antigen presenting ability of SPMs and LPMs *in vitro*, peritoneal cavity cells were collected from naïve C57BL/6 mice. CD3^+^ T cells, CD19^+^ B cells, CD11c^+^ dendritic cells (DCs), Ly6G^+^ neutrophils, and CD11b^lo^SSC^hi^ eosinophils were excluded, and then CD11b^+^ macrophages were identified. Among these macrophages, CD11b^+^F4/80^lo^ SPMs and CD11b^hi^F4/80^hi^ LPMs were distinguished (Fig. 1A) and separately sorted with purity more than 90% (Fig. 1B). CD4^+^CD62L^hi^CD44^dull^ naïve T cells isolated from OT-II Tg mice were labeled with carboxyfluorescein succinimidyl ester (CFSE) to observe T cells proliferation. After the coculture of T cells with SPMs loaded with 1 µM of pOVA for 5 days, approximately 30% of T cells proliferated (Fig. 1C). The percentages of proliferated T cells were dependent on the concentration of pOVA (Fig. 1D). By contrast, T cells did not proliferate after coculture with LPMs loaded with any of the doses of pOVA examined (Fig. 1C,D). These results indicate that SPMs have ability to prime naïve CD4^+^ T cells.

### DNAM-1 is strongly expressed on SPMs

**Figure 2:**
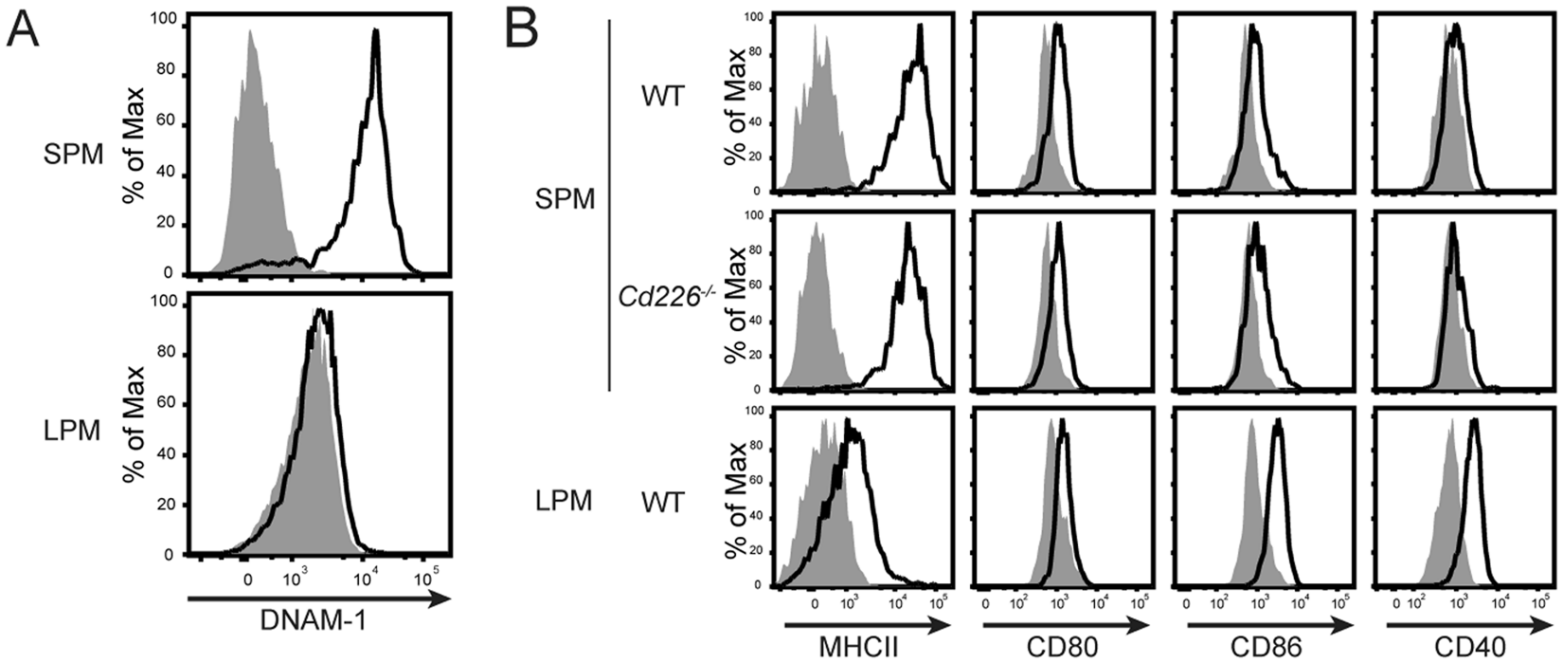
DNAM-1 (CD226) is strongly expressed on SPMs. (**A**) DNAM-1 expression on SPMs and LPMs detected by anti-DNAM-1 mAb. (**B**) Expression of costimulatory molecules involved in antigen presentation on WT SPMs, *Cd226*^−/−^ SPMs and LPMs. Open histograms indicate specific mAb and shaded histograms indicate isotype controls. Data is representative of over two independent experiments.

To find functional molecules on SPMs, we compared gene expression between the mouse peritoneal macrophage subsets CD115^+^B220^−^MHCII^+^F4/80^lo^ cells and CD115^+^B220^−^MHCII^−^F4/80^hi^ cells by using the microarray data of the Immunological Genome (Immgen) Project^20^, (http://www.immgen.org); the definitions of these subsets share similar features with the definitions of SPM and LPM, respectively^3^. Expression of the *Cd226* gene in MHCII^+^F4/80^lo^ cells was 58 times more than that in MHCII^−^F4/80^hi^ cells (Immgen Population Comparison; MF_II + 480lo_PC vs. MF_II-480hi_PC). In addition, analysis using the Gene Skyline browser of Immgen revealed that *Cd226* gene expression was especially high in MHCII^+^F4/80^lo^ cells compared with other macrophage subsets, monocytes, and neutrophils (Immgen Gene Skyline; gene symbol = *Cd226*). Although the definitions of the above mouse peritoneal macrophage subsets share similar features with the definitions of SPM and LPM, there are several differences between them, among which the most important is the exclusion of CD11c^+^ cells in the definition of SPMs. Therefore, we examined DNAM-1 expression on SPMs and LPMs; we found that DNAM-1 protein was strongly expressed on SPMs, but not on LPMs (Fig. 2A). However, the proportions of SPM in wild-type (WT) and DNAM-1-deficient (*Cd226*^−/−^) mice were comparable (Supplementary Fig. 1A). CD11c^+^ peritoneal DCs also express DNAM-1, but at a medium level compared to SPMs (*data not shown*). Thus, we revealed uniquely high expression of DNAM-1 on SPMs.

Consistent with previous reports^3^, SPMs expressed higher levels of MHCII than did LPMs (Fig. 2B). The expression levels of both MHCII molecules and costimulatory molecules were comparable between WT mice and *Cd226*^−/−^ mice (Fig. 2B), indicating that DNAM-1 had no effect on expression levels of either MHCII or costimulatory molecules.

### DNAM-1–CD155 interaction is involved in costimulation of CD4^+^ T cells

**Figure 3:**
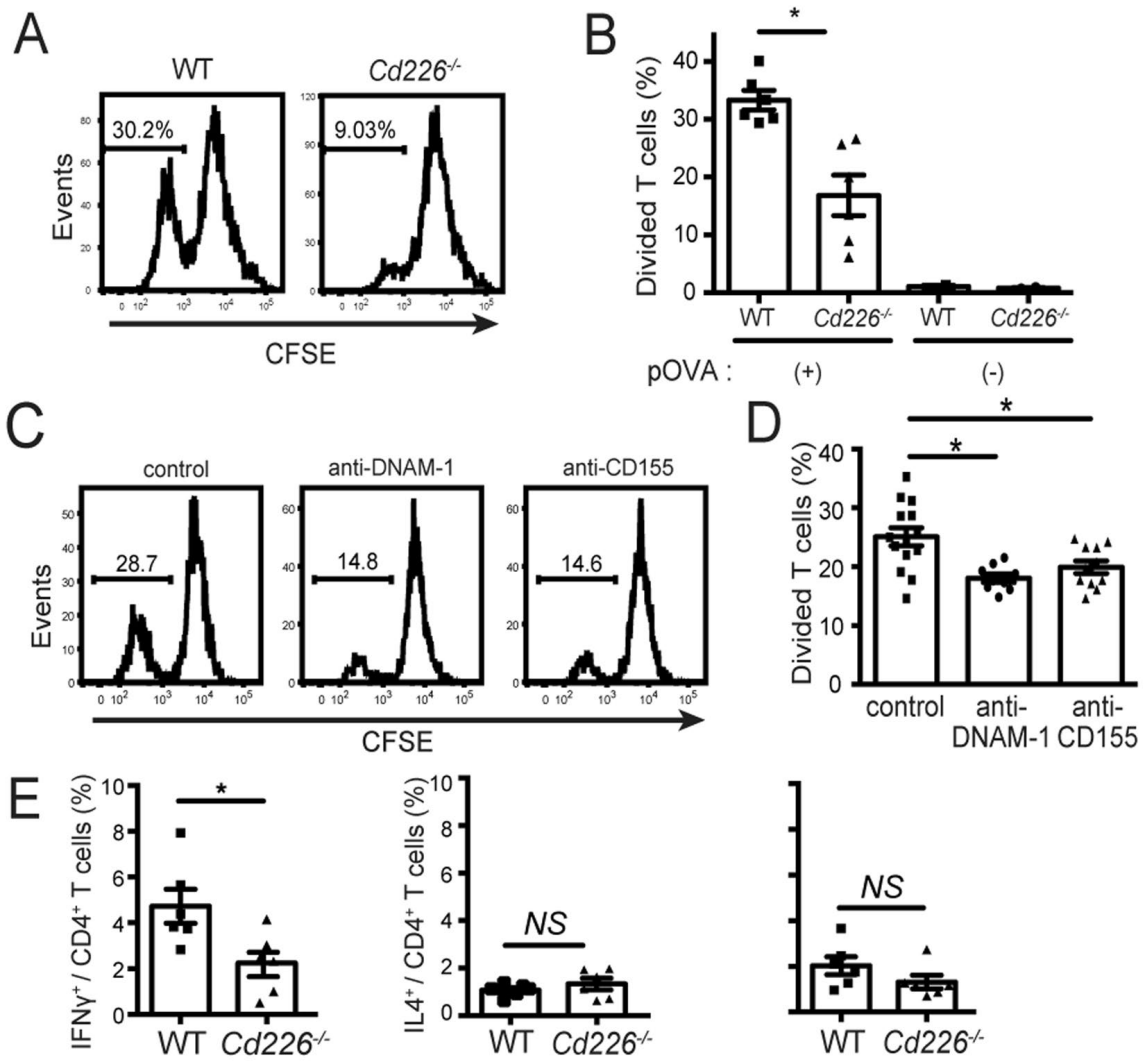
DNAM-1–CD155 interaction is involved in antigen presentation by SPMs. (**A**,**B**) Naïve CD4^+^ T cells isolated from OT-II Tg mice were labeled with CFSE and cocultured with SPMs isolated from WT or *Cd226*^−/−^ mice in the presence of 1 µM pOVA. T cells proliferation was assessed by CFSE dilution as mentioned in Fig. 1C,D. Data are representative of four independent experiments. (**C**,**D**) Naïve CD4^+^ T cells were cocultured with WT SPMs as mentioned in (**A**,**B**). Either CD4^+^ T cells were pretreated with anti-CD155 mAb or SPM were pretreated with anti-DNAM-1. Anti-mouse CD155 mAb (TX56, rat IgG2a)^19^ and anti-mouse DNAM-1 mAb (TX42)^18^ were generated in our laboratory. T cells proliferation was assessed by CFSE dilution as mentioned in Fig. 1C,D. Data are representative of two independent experiments. Histograms show representative data (**A**,**C**) and bar graph shows the average of three wells after culture with pOVA (**B**,**D**). (**E**) After 6 days of coculture as mentioned in (**A**,**B**), the cells were restimulated by phorbol 12-myristate 13-acetate (PMA) and ionomycine for 4 hrs, then stained with anti-CD4 to distinguish CD4^+^ T cells with SPMs, and intracellularly stained with anti-IFNγ, anti-IL4 and anti-IL17 to evaluate Th differentiation of CD4^+^ T cells by flow cytometry analysis. Bar graph shows the average of three wells after culture with or without pOVA (E). Data are representative of two independent experiments. SEM, ^*^P < 0.05. *NS*, not significant.

Since SPMs are able to prime naïve CD4^+^ T cells, we examined whether DNAM-1 on SPM is involved in this function. We first compared the CD4^+^ T cells priming abilities of SPMs isolated from WT mice and *Cd226*^−/−^ mice. The frequency of proliferated T cells after coculture with pOVA–loaded *Cd226*^−/−^ SPMs was significantly lower than that with WT SPMs (Fig. 3A,B), suggesting that DNAM-1 is involved in T cell priming by SPMs. We next examined whether DNAM-1 is involved in antigen uptake or processing by SPMs; however, both genotypes of SPMs showed comparable levels of phagocytosis and antigen processing (Supplementary Fig. 1B,C).

Because we previously reported that CD155, a DNAM-1 ligand, on CD4^+^ T cells plays an important role in costimulation of T cells and promote Th1 differentiation independent of IL-12^21^, we supposed the ligation of DNAM-1 on SPMs with CD155 on T cells induces T cells proliferation. Then, we compared the T cells responses primed by SPMs in the presence of anti-CD155 Ab, anti-DNAM-1 neutralizing Ab or isotype-matched control Ab. The frequency of T cells proliferation was impaired after either coculture of anti-DNAM-1 Ab-pretreated SPMs with CD4^+^ T cells or SPMs with anti-CD155 Ab-pretreated CD4^+^ T cells compared to isotype control (Fig. 3C,D). These results indicate that the interaction between DNAM-1 on SPMs and its ligand CD155 on T cells is indispensable for the function of DNAM-1 in SPM-mediated antigen specific T cells priming.

Next, we examined cytokine production from CD4^+^ T cells after being primed by SPMs from WT mice or *Cd226*^−/−^ mice. The frequency of IFNγ^+^ CD4^+^ T cells after coculture naïve CD4^+^ T with pOVA–loaded *Cd226*^−/−^ SPMs was significantly lower than that with WT SPMs, while frequency of IL-4^+^ and IL-17^+^ CD4^+^ T cells were compatible (Fig. 3E). On the other hand, IL-12 production was compatible between WT and *Cd226*^−/−^ SPMs (Supplementary Fig. 1D), indicating that the reduced IFNγ production from *Cd226*^−/−^ CD4^+^ T cells is independent of IL-12 production from SPMs. These results indicate that SPMs activate naïve CD4^+^ T cells and then promote Th1 differentiation in the dependence on costimulatory signal generated by the interaction of DNAM-1 on SPMs and CD155 on T cells.

### DNAM-1 on SPMs contributes to T cells proliferation in the draining lymph nodes after intraperitoneal immunization

**Figure 4:**
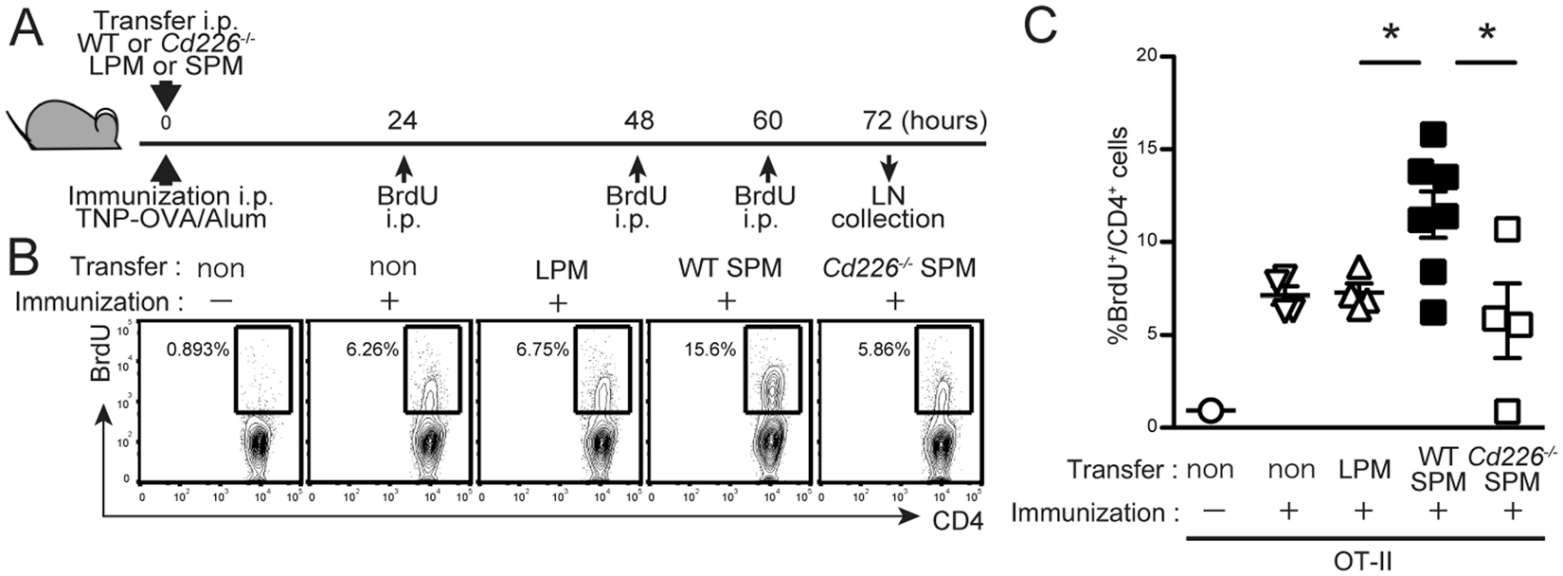
DNAM-1 on SPMs contributes to T cells proliferation in mediastinal lymph nodes after immunization. (**A**–**C**) OT-II Tg recipient mice were transferred i.p. with 4 × 10^4^ of LPMs or WT or *Cd226*^−/−^ SPMs and immunized i.p. with TNP-OVA/Alum and cells proliferation were traced by injecting BrdU as shown in Materials and Methods. After 72 hrs, mediastinal LNs were collected; the frequencies of dividing CD4^+^ T cells were defined as the percentage of BrdU^+^ cells in CD3^+^CD4^+^ cells. Nuclei were stained with APC-conjugated anti-BrdU mAb by using a BrdU Flow Kit (BD Biosciences). Schematic view of the experiment (**A**). Representative plots of BrdU staining (**B**). Scatter plot showing data combined from two independent experiments (**C**); SEM, ^*^P < 0.05.

To investigate whether DNAM-1 on SPMs is involved in CD4^+^ T cell priming *in vivo*, OT-II Tg mice were subjected to adoptive cell-transfer i.p. with 4 × 10^4^ SPMs or LPMs sorted from WT or *Cd226*^−/−^ donor mice; at the same time, immunized i.p. with TNP–OVA/Alum. On day 3, CD4^+^ T cells proliferation in the mediastinal lymph nodes (LNs, draining LNs from peritoneal cavity^22^) were analyzed by BrdU incorporation (Fig. 4A). T cells proliferation was significantly greater in SPM-transferred mice than in LPM-transferred mice or control mice without cell transfer (Fig. 4B,C). However, this enhanced proliferation of T cells was abolished in mice subjected to cell transfer with *Cd266*^−/−^ SPMs (Fig. 4B,C). To confirm the involvement of CD155 on T cells in CD226-mediated T cell proliferation, we used *Cd155*^−/−^ OT-II mice. When WT or *Cd266*^−/−^ SPMs were transferred into *Cd155*^−/−^ OT-II mice, the proliferation of both genotypes of SPM showed comparable (Supplementary Fig. 2). Together, these results indicate that the interaction between DNAM-1 on SPMs and CD155 on CD4^+^ T cells contributes to T cells priming in the draining lymph nodes after antigen immunization via the peritoneal cavity.

### DNAM-1 on SPMs contributes to the germinal center reaction in mediastinal lymph nodes after immunization

**Figure 5:**
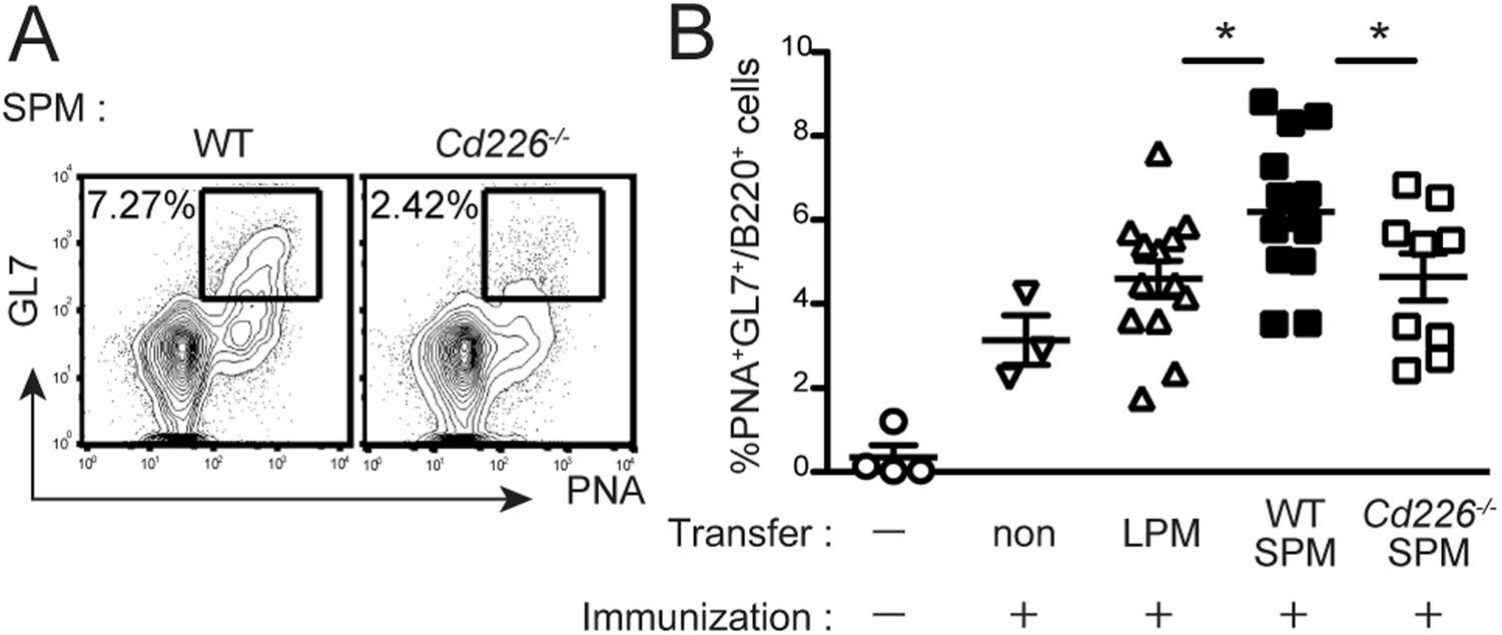
DNAM-1 on SPMs contributes to the GC reaction in mediastinal lymph nodes after immunization. (**A**,**B**) LPMs and SPMs were isolated from WT or *Cd226*^−/−^ donors, and 4 × 10^4^ cells were transferred i.p. into *Cd226*^−/−^ recipient mice. At the same time, the recipient mice were immunized i.p. with TNP–OVA in alum adjuvant. Seven days after immunization, mediastinal lymph nodes were collected; the cells were stained with GL7 mAb, PNA, and anti-B220 mAb, and the frequencies of GC B cells (defined as the percentages of GL-7^+^PNA^+^cells in B220^+^ B cells) were determined by flow cytometry. Representative plots of staining (B220^+^ gate) from SPM-transferred mice (**A**). The scatter plot shows data combined from four independent experiments (**B**) error bars indicate SEM. ^*^P < 0.05. One outlier was omitted from *Cd226*^−/−^.

To investigate whether DNAM-1 on SPMs affects the humoral immune response, we analyzed the GC reaction in the mediastinal lymph nodes after immunization. WT or *Cd226*^−/−^ SPMs were transferred i.p. into *Cd226*^−/−^ recipient mice. The mediastinal lymph nodes were harvested 7 days after immunization and analyzed for frequency of occurrence of GC B cells (defined as GL7^+^PNA^+^B220^+^ cells). The GC reaction was greater in SPM-transferred mice than in LPM-transferred mice or control mice without cell transfer (Fig. 5B). Furthermore, the enhanced GC reaction was abolished in mice that had received *Cd266*^−/−^ SPMs (Fig. 5A,B). Together with the data in Figs 4 and 5, these results suggest that DNAM-1 expressed on SPMs in the peritoneal cavity is involved in T cell priming and the humoral immune response in the draining lymph nodes after immunization.

Taken together, our results show that SPMs are functionally different from LPMs. They also show that DNAM-1 on SPMs plays an important role in priming T cells in the draining lymph nodes of the peritoneal cavity against antigens present in this cavity.

## Discussion

Previous reports demonstrated that peritoneal macrophages engulf pathogens, induce neutrophils recruitment into the peritoneal cavity and thus play important roles in the control of infections^23–25^; the macrophages analyzed in these reports are likely LPMs on the basis of their surface markers. In contrast, we revealed that SPMs, a newly defined subset of peritoneal macrophages, but not LPMs, play an important role in adaptive immunity against i.p. injected antigen. Note that SPMs are distinct from peritoneal DCs; the former have much less expression of CD11c and different levels of expression of CD11b and F4/80^3^. Some reports have defined two populations of mouse peritoneal macrophages, MHCII^lo^F4/80^hi^ and MHCII^hi^F4/80^lo^ ^6,26,27^. MHCII^lo^F4/80^hi^ cells in these reports likely correspond to LPMs, considering the reported gating strategy used in the flow cytometry analysis. In contrast, MHCII^hi^F4/80^lo^ cells in these reports appear to be mixtures of SPMs and DCs; DCs have a CD11b^mid^MHCII^hi^F4/80^−^ phenotype (1), which is somewhat similar to the CD11b^+^MHCII^hi^F4/80^lo^ phenotype of SPMs, but DCs can be distinguished from SPMs by their stronger expression of CD11c, which was not assessed in these reports. Therefore, we suspect that the antigen presenting ability of MHCII^hi^F4/80^lo^ cells, which has been reported previously^27^, reflects the function of DCs. Our finding demonstrates direct evidence for the antigen presenting ability of SPMs.

We showed that SPMs were involved in T cell priming and GC B cell development *in vivo*. Because CD4^+^ helper T cells are essential for GC maintenance^28^, our data indicate that SPMs are involved in CD4^+^ T cell proliferation in response to antigen immunization, which in turn enhances the humoral immune response. Given that peripheral tissue immunization with antigen in adjuvant induces antigen uptake by local DCs and priming of T cells in the draining lymph nodes^29^, it is speculated that SPMs take up antigen in the peritoneal cavity, migrate into the draining lymph nodes, and present antigen to naïve CD4^+^ T cells^3^. In support of this idea, we showed in this report that SPMs are able to uptake and process antigens *in vivo*, consistent with previous reports^3,7,9^. Moreover, Ghosn *et al*. reported that half of SPMs express the lymphocyte homing receptor CD62L; they also suggested that SPMs migrate into lymph nodes^3^. Although our data suggest that SPMs migrate into the mediastinal lymph nodes and present antigen to T cells, further investigations are required to demonstrate the migration of SPMs into draining lymph nodes.

We showed here that DNAM-1 is expressed on SPMs at an exclusively high level among peritoneal macrophages. A recent report by Kim *et al*. has demonstrated that DNAM-1 served as a distinct surface marker of MHCII^+^ resident peritoneal macrophages compared to F4/80^+^ counterpart^30^. Because MHCII^+^ resident peritoneal macrophages and SPMs share MHCII molecule as a surface marker, both populations in the peritoneal cavity may overlap each other. More interestingly, we revealed that DNAM-1 enhances the antigen-specific CD4^+^ T cell priming by SPMs. Whereas the function of DNAM-1 as an activating receptor on T cells and NK cells had been well studied^10–13,16–19^, its function in macrophages had not yet been reported. Our report provides new understanding that DNAM-1 is one of the molecules characterizing particular functions of heterogeneous macrophage subsets.

DNAM-1-deficient SPMs showed markedly lower antigen presentation ability than WT SPMs. In addition, T cells proliferation was impaired when the T cells were pretreated with anti-CD155 neutralizing Ab *in vitro* and mice deficient in CD155 transferred with SPM were analyzed. Consistent with our recent findings that CD155 mediated a costimulatory signal in naïve CD4^+^ T cells and induce Th1 differentiation^21^, these results indicate that interaction between DNAM-1 and its ligand, CD155, is indispensable for the function of DNAM-1 in the antigen presentation of SPMs. Whereas DNAM-1 acts as a signaling molecule on T cells and NK cells^10,31^, it is unclear whether DNAM-1 on SPMs mediates an activating signal in SPMs and induces changes in the function of SPMs. Even though we do not exclude the possibility of intracellular signaling of DNAM-1 on SPMs, our data suggest that DNAM-1 on SPMs interacts with CD155 expressed on CD4^+^ T cells, upregulates T cells proliferation and promotes Th1 differentiation via CD155-mediated costimulatory signals.

We propose that SPMs are functionally distinct from LPMs and that the DNAM-1–CD155 axis plays an important role in costimulation of antigen-specific CD4^+^ T cells. In this context, the exclusively high expression of DNAM-1 on SPMs characterizes the function of SPMs among heterogeneous subsets of peritoneal macrophages.

## Methods

### Mice

C57BL/6 mice were purchased from Clea Japan (Tokyo, Japan). *Cd226*^−/−^ mice were generated as described previously^18^. Ovalbumin peptide (OVA_323–339_)-specific, MHC class II I-A^b^-restricted αβ T cells receptor (TCR) transgenic (OT-II Tg) mice were originally constructed as described by Barnden *et al*.^32^. *Cd155*^−/−^ mice were kindly provided by Dr. Günter Bernhardt^33^. *Cd155*^−/−^ OT-II mice were generated in our lab by crossing *Cd155*^−/−^ and OT-II mice. All mice used were 8–16-week-old females or males and on the C57BL/6 background. All animal experiments were performed humanely after receiving approval from the Animal Ethics Committee of the Laboratory Animal Resource Center, University of Tsukuba, and in accordance with Fundamental Guideline for Proper Conduct of Animal Experiment and Related Activities in Academic Research Institutions under the Jurisdiction of the Ministry of Education, Culture, Sports, Science and Technology.

### Flow cytometry analysis and isolation of SPMs

Peritoneal cells were harvested and then stained with the Abs described below: FITC-anti-mCD3 (145-2C11), CD19 (1D3), CD11c (HL3), Ly6G (1A8), and CD40 (HM40-3), PE-anti-mCD11b (M1/70), PE-Cy7-anti-mCD4 (RM4–5), biotin-anti-mCD80 (16-10A1), CD86 (PO3) mAbs, isotype-matched control Ab, PE-Cy7 and V450-streptavidin, (BD Biosciences, San Jose, CA, USA); Alexa647-anti-mF4/80 mAb (C1:A3-1) (AbD Serotec, Kidlington, UK); PE-Cy7-anti-mI-A^b^ mAb (AF6–120.1) (BioLegend, San Diego, CA, USA); and anti-mDNAM-1 mAb (Clone, TX42. Isotype, rIgG2a) generated in our laboratory^18^ and conjugated with biotin; for intracellular staining, PE-anti-mIL4 (11B11) and Alexa647-anti-mIL17 (TC-11–18H10) (BD Biosciences) and FITC-anti-mIFNγ (XMG1.2) (TONBO Biosciences, San Diego, CA, USA) mAbs.

Propidium iodide was used to identify and exclude dead cells. Sample acquisition was performed by using a FACSCallibur or FACSFortessa cell analyzer (BD Biosciences). FlowJo software (Tree Star, Ashland, OR, USA) was used for data analysis. LPMs and SPMs were sorted on a FACSAria sorter (BD Biosciences).

### Isolation of naïve CD4^+^ T cells

CD4^+^ T cells were isolated from the spleens of OT-II Tg mice by using anti-mCD4 MACS technology (Miltenyi Biotec, Auburn, CA, USA) according to the manufacturer’s instructions. Cells were stained with PE-Cy7-anti-mCD4 (RM4–5), allophycocyanin (APC)-anti-mCD62L (MEL-14), and APC-Cy7-anti-m/hCD44 (IM7) mAbs (BD Biosciences), and CD62L^hi^CD44^dull^CD4^+^ naïve T cells were sorted by using a FACSAria cell sorter.

### T cell proliferation assay

Isolated naïve CD4^+^ T cells were labeled with CFSE. T cells (2 × 10^4^ cells) were cocultured with the same number of LPMs or SPMs in the presence of 0.25, 0.5, or 1 µM of OVA_323–339_ peptide (pOVA). On day 5, cells were stained with PE-conjugated anti-mouse CD11b mAb, and then CFSE dilution was measured by using a FACSCalibur cell analyzer (BD Bioscience).

### Immunization and cell transfer assay

For conjugating with trinitrophenyl (TNP), OVA (Sigma-Aldrich, St. Louis, USA) was incubated with picrylsulfonic acid (pH 8.5) overnight at 4 °C and then dialyzed with PBS; the average number of TNP per OVA molecule was 7.9. Mice were immunized i.p. with 35 µg of TNP–OVA emulsified with 100 µl of 2% alum adjuvant (InvivoGen, San Diego, CA, USA) (TNP-OVA/Alum). For the *in vivo* proliferation assay, the mice were injected i.p. with 200 µl of 10 mg/ml 5-bromo-2′-deoxyuridine (BrdU) in saline (2 mg) at 24, 48, and 60 h after immunization. For germinal center (GC) staining, mediastinal lymph nodes were collected 7 days after immunization; cells were stained with PE-conjugated anti-mouse B220, Alexa647-conjugated GL7 mAb (BD Biosciences), and biotinylated peanut agglutinin (PNA; Vector Laboratories, Burlingame, CA), followed by FITC-conjugated streptavidin. Cells were analyzed with a FACSCalibur cell analyzer (BD Biosciences).

### Phagocytosis assay

Mice were i.p. injected with 200 μg of pHrodo Red-conjugated *Staphylococcus aureus* (A10010, Invitrogen, Carlsbad, CA, United States). Two hours later, peritoneal cells were collected and SPMs, which was identified as Zombie^−^, Lin (CD3, CD19, CD11c, Ly6G, and Ly6C)^−^, CD11b^hi^, F4/80^lo^ cells, were analyzed for pHrodo fluorescence by flow cytometry (FACSAria Special Order Research Products (SORP) sorter (BD Biosciences)).

### Antigen processing assay

One hundred μg of BODIPY-labelled dye quenching Ovalbumin (DQ-OVA, D12053, Invitrogen, Carlsbad, CA, United States) emulsified with 100 µl of 2% alum adjuvant was i.p. injected into mice. One hour later, peritoneal cells were collected and SPMs, which was identified as described above, were analyzed for a green fluorescence derived from digestion products by flow cytometry (FACSAria Special Order Research Products (SORP) sorter (BD Biosciences)).

### *In vitro* IL-12 production

Isolated naïve CD4^+^ T cells (2 × 10^4^ cells) were cocultured with the same number of SPMs in the presence of 1 µM of pOVA. At 0, 20 and 40 h, the culture supernatant was measured for IL-12 content by ELISA.

### Statistical analysis

Statistical analyses were performed by using the unpaired two-sided Student’s *t*-test (GraphPad Prism  7, GraphPad Software, La Jolla, USA). *P* values (*P*) less than 0.05 were considered statistically significant.

## Electronic supplementary material

Supplementary Information

## Data Availability

No datasets were generated or analysed during the current study.
