# Supplementary material for: Selective DNAM-1 expression on small peritoneal macrophages contributes to CD4+ T cell costimulation
Source: Sci Rep. 2018 Oct 12;8:15180. doi: 10.1038/s41598-018-33437-4 (PMC6185969; doi:10.1038/s41598-018-33437-4)
Supplement: Supplementary file 1 — Supplementary Information [file 41598_2018_33437_MOESM1_ESM.docx]

**Article**

**Selective DNAM-1 expression on small peritoneal macrophages contributes to CD4^+^ T cell costimulation**

Eri Takenaka^1,#^, Anh Van Vo^1,2,#^, Yumi Yamashita-Kanemaru^1^, Akira Shibuya^1,3^, Kazuko Shibuya^1,*^

**Supplementary Information**

**
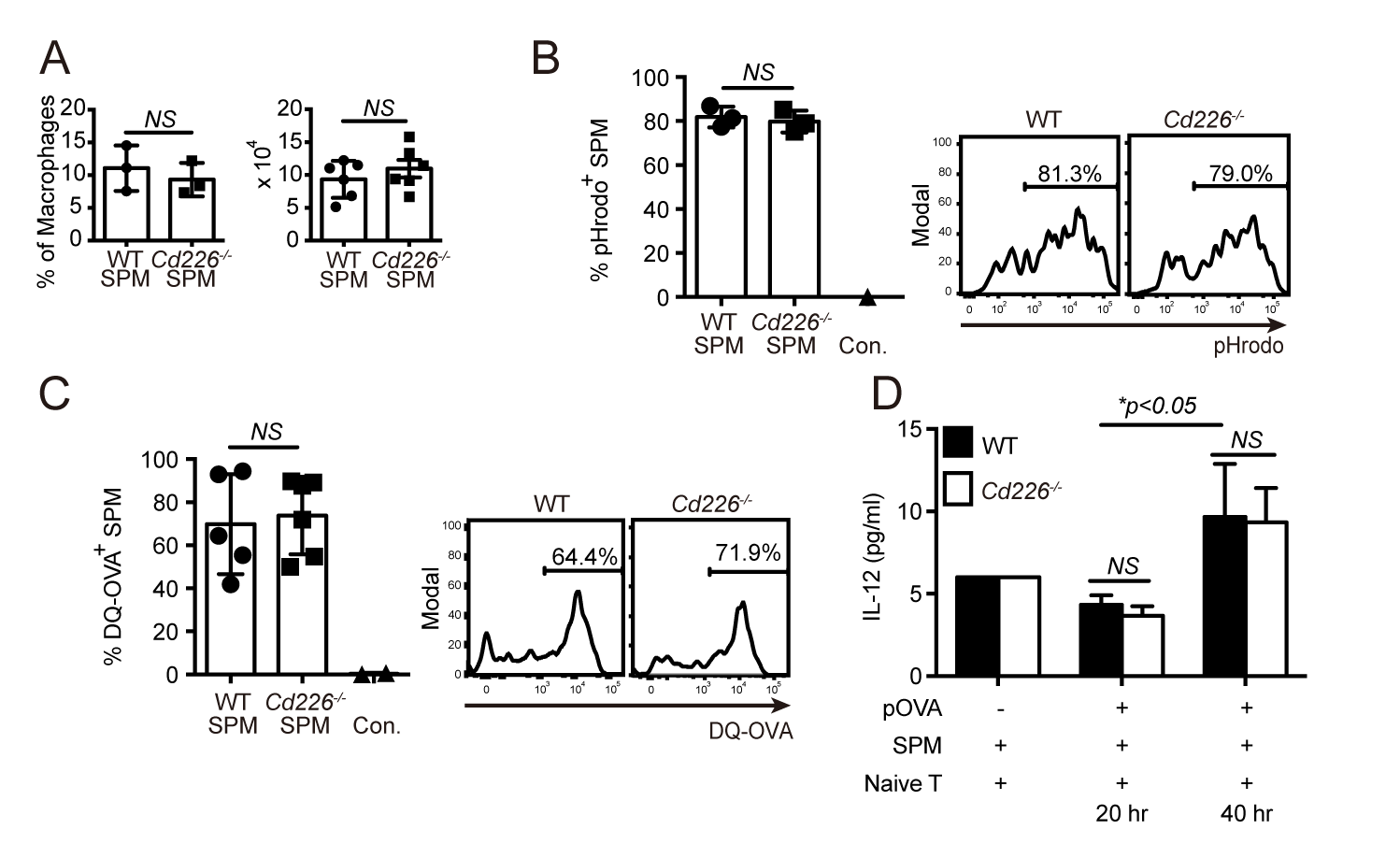
**

**Supplementary Fig. 1. DNAM-1 is not involved in the number of SPMs, antigen phagocytosis and processing and cytokine production by SPMs.**

(A) The proportion (n=3) and number (n=6, combined from two independent experiments) of WT and *Cd226^-/-^* SPMs in the peritoneal cavity. Gating strategy of SPMs was shown in Fig. 1*A*.

(B) Antigen phagocytosis by SPMs. WT or *Cd226^-/-^* mice were i.p. injected with pHrodo Red-conjugated *Staphylococcus aureus*. Two hours later, peritoneal cells were collected and SPMs were analyzed for antigen uptaking (pHrodo^+^ SPMs) by flow cytometry. Data are representative from two independent experiments.

(C) Antigen processing by SPMs. DQ- OVA emulsified with alum adjuvant was i.p. injected into WT or *Cd226^-/-^* mice. One hour later, peritoneal cells were collected and SPMs were analyzed for antigen processing (DQ-OVA^+^ SPMs) by flow cytometry. Data are representative from two independent experiments.

(D) The *in vitro* production of IL-12 by SPMs. The pOVA-loaded WT or *Cd226^-/-^* SPMs were cocultured with naïve CD4^+^ T cells for 20 or 40 h, and then the culture supernatants were measured for IL-12 by ELISA. Error bars indicate SEM. **P* < 0.05.

**
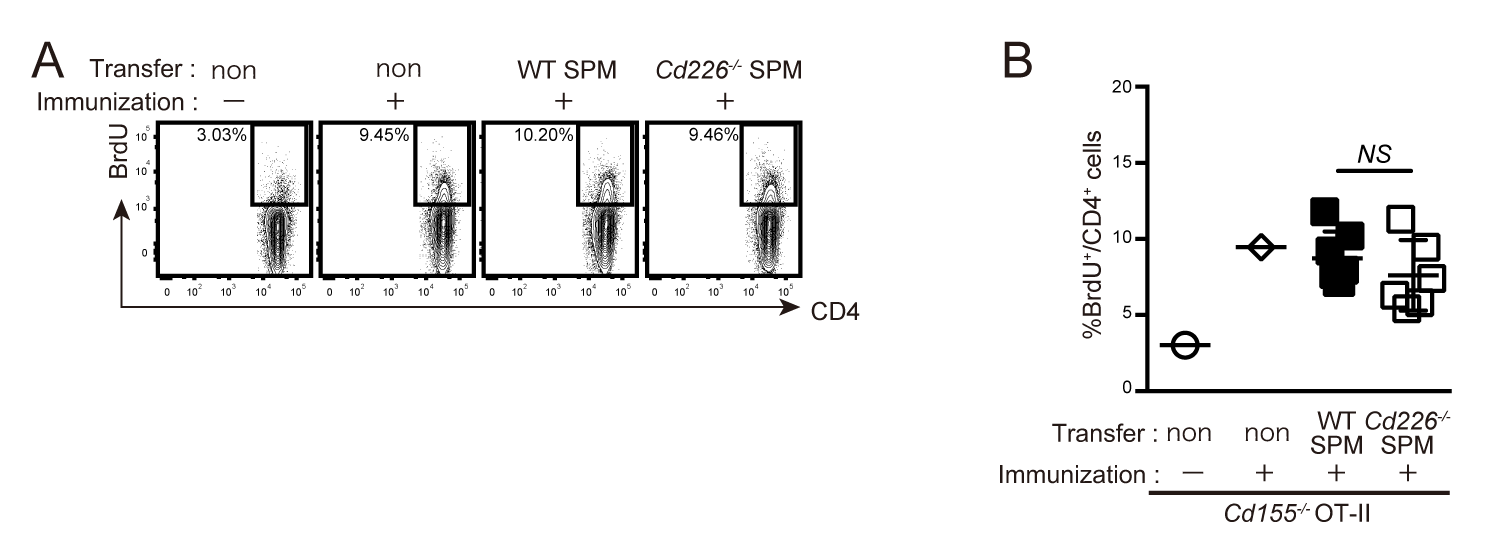
**

**Supplementary Fig. 2. Involvement of CD155 in CD226-mediated costimulation of CD4^+^ T cells.**

*Cd155^-/-^* OT-II mice were transferred i.p. with WT or *Cd226^-/-^* SPMs and immunized i.p. with TNP-OVA/Alum and cells proliferation were traced by injecting BrdU. Mice were analyzed, described in Fig. 4. Representative plots of BrdU staining (A). Scatter plot showing representative data from two independent experiments (B).
